# Principles of Botany

**Published:** 1853-07

**Authors:** 


*Principles of Botany*, as exemplified in the Cryptogamia, for the use of Schools and Colleges. By HARLAND COULTAS. Philadelphia: LINDSAY & BLAKISON, 1853.

IT is a peculiarity of the profession of medicine, that its science comprehends the whole field of the natural sciences. It is a vast temple, its dome bathed in the pure life-giving air of heaven, its foundation laid deep in the earth, its walls built of the solid rock, its altars decorated with gems from every clime, and precious ores from every mine, its tapestry the green foliage gathered from every forest, and its carpeting the flowers from a thousand hills and a thousand vales. This building of beauty and grandeur is, or should be, the home of the physician; air, earth, mineral plant and flower, are all his to use as instruments for alleviating the suffering of the race and for combating death. It is true, we may not, each one of us, have examined carefully and understood perfectly all the different branches of the one great science, we may not each have ministered at all the altars of the temple, but it is our boast, that our science is a unity, that at every altar the same name is invoked and the same rite performed. In true medical sciences there are no schools or sects; there can be none, from the very necessity of the case. It is only when we come into the region of theory and speculation; when, leaving the known, we pass into the unknown, and see all things by a dim intellectual twilight, that we may differ in our conjectures of the realities of our surroundings.

We have been led, strangely, perhaps, to the above train of reflections, by the little work whose title stands at the head of this article. It is an introduction to the science of botany, rather than a treatise. The question is asked, how does nature, the architect of the floral temple, form this green leaf and beautiful blossom? What are the modes in which she works for the accomplishment of such varied and beautiful results? Although we cannot answer, satisfactorily, this question, we can unfold the structure, observe the relation, and study the functions of parts. In the first chapter the author treats of cellular tissue, of the primitive and modified forms of cells. In the second chapter the subject of woody and vascular tissue is discussed. In the third, the development and functions of cells. This is an interesting chapter and completes the first part.

The second part treats of the compound organs of plants, the cryptogamia or flowerless plants. The whole work is interesting, and fulfills the intention of the writer, by exciting a desire to know more of the phenomena of life. We agree with the author, "that the study of the simple plants ought to take the precedence of those whose organization is more complex and intricate, as being the simplest expression of the laws of vegetable life.

